# Enhancing the chemotherapy effect of Apatinib on gastric cancer by co-treating with salidroside to reprogram the tumor hypoxia micro-environment and induce cell apoptosis

**DOI:** 10.1080/10717544.2020.1754528

**Published:** 2020-05-13

**Authors:** Zhandong Zhang, Wei Yang, Fei Ma, Qi Ma, Bin Zhang, Yonglei Zhang, Yingqiang Liu, Hongxing Liu, Yawei Hua

**Affiliations:** Department of General Surgery, Affiliated Tumor Hospital of Zhengzhou University, Henan Cancer Hospital, Henan, Zhengzhou

**Keywords:** drug-resistance, hypoxic microenvironment, nanoparticles, tumor targeting, drug delivery

## Abstract

Hypoxic microenvironment commonly occurred in the solid tumors considerably decreases the chemosensitivity of cancer cells. Salidroside (Sal), the main active ingredient of *Rhodiola rosea*, was shown to be able of regulating the tumor hypoxia micro-environment and enhancing the chemotherapeutic efficacy of drug-resistant cancer. Therefore, in this study, the Sal was co-loaded with Apatinib (Apa) by the PLGA-based nanoparticles (NPs) to improve the chemosensitivity of gastric cancer cells. Additionally, to improve the drug delivery efficacy, the tumor-homing peptide (iVR1 peptides) was further decorated on the surface of NPs. The tumor targeting ability of the peptides-functionalized nanoparticles (iVR1-NPs-Apa/Sal) was evaluated by *in vitro* and *in vivo* experiments. As the obtained results revealed that the iVR1-NPs-Apa/Sal displayed excellent tumor affinity than the unmodified ones (NPs-Apa/Sal), which in turn resulted in more efficient of anti-proliferation of gastric cancer cells and anti-tumor effect *in vivo*. In addition, compared with the cells or tumor-bearing mice only treaded by monotherapy of Apa, the cells or mice received combinational treatment of Apa and Sal showed obvious lower rate of growth, invasion, and migration or tumor growth and progress. Underlying mechanisms were further investigated and it was revealed that the anti-gastric cancer effect of Apa was signally improved by Sal through down-regulation the proliferation factors and increase the pro-apoptotic genes, as well as reprograming the tumor hypoxia micro-environment. In a word, the study showed that the Sal was able of improving the chemosensitivity of gastric cancer to Apa and the iVR1-NPs-Apa/Sal was capable of realizing highly efficient of tumor-targeting drug delivery.

## Introduction

Giving the rapid increased incidence and deteriorate rate, the gastric cancer (GC) has been the fifth frequently diagnosed cancer type and leading cause of cancer deaths (Chen et al., 2017; Bray et al., 2018). Besides, the lack of obvious symptoms and absence of sensitive and specific biomarkers resulted to most of patients with GC were diagnosed at the late stage (Mikami et al., 2017). Statistical data revealed that the overall survival of patients diagnosed with GC is extremely poor and further decreased after tumor metastasis (Jiang et al., 2018). To date, chemotherapy remains one of the major modalities of GC treatment. However, the clinical success of chemotherapy signally impaired by the emergence of drug-resistance due to overexpression of multidrug resistance 1 (MDR1) (Chang 2011; Kunjachan et al., 2013).

As the receptor tyrosine kinase inhibitor, Apatinib (Apa) is able of selectively targets the intracellular ATP binding site of VEGFR-2 and has shown relative excellent efficacy in treatment of GC with metastasis (Pera et al., 2012; Bajetta et al., 2014). Previous clinical trials revealed that Apa has prolonged the median overall survival and progression-free survival by 55 days and 25 days, respectively, compared with the placebo (Geng and Li, 2015). However, the initially Apa-sensitive of GC was greatly down-regulated by emergence of acquired drug-resistance during treatments (Teng et al., 2018). The acquired cancer resistance was contributed by multiple factors such as the DNA damage, drug efflux, cancer stem cells, and alterations in drug targets (Ma et al., 2018).

Beside the acquired resistant mechanisms, there existed as well as the intrinsic cancer resistance caused by the extremely complex tumor microenvironment (Ma et al., 2018). Hypoxic microenvironment within solid tumors has been supposed to be one of the primary predisposing factors of rug resistance (Vaupel, 2004; Baran and Konopleva, 2017). In contrast to the normal tissues, the concentration of oxygen is markedly reduced in many solid tumors due to the rapid proliferation of cancer cells and abnormalities of blood vessels (Koh and Powis, 2012; Baran and Konopleva, 2017; Yu et al., 2019). Hypoxia regulates drug resistance in diverse types of solid cancer, including osteosarcoma (Wang et al., 2018), multiple myelomas (Nakagawa et al., 2018), and hepatocellular carcinoma (Yu et al., 2019). It was previously shown that the hypoxia inducible factor (HIF) facilitates the formation of hypoxic microenvironment through regulation of transcription by hypoxia-inducible factor 1 (HIF-1) and HIF-2 (Martin et al., 2011; Hahne et al., 2018).

Salidroside (Sal) represents the main active ingredient of *Rhodiola rosea* and has been shown with multiple biological activities, such as relieving high altitude sickness, replenishing vital energy, and protecting blood vessels (Wang et al., 2009; Zheng et al., 2011). Anticancer effects of Sal has also been confirmed in the bladder tumor models (Gui et al., 2017). In this study, the Sal showed excellent ability to inhibit the progress of bladder tumor by activating the ERS-dependent autophagy (Gui et al., 2017). Furthermore, other study showed that Sal was able of suppressing the activation of HIF-1α signaling pathway and therefore signally improving the chemosensitivity of drug-resistant cancer cells (Qin et al., 2018). Based on this, combination therapy of cancer with chemotherapeutics plus Salid may holds great potential in enhancing the treatment efficacy of drug-resistant cancers.

In this study, to improve the drug delivery efficacy and therapy effect of Apa, it was co-loaded with Sal by the PLGA-based nanoparticles (NPs-Apa/Sal). To achieve the goal of releasing drugs at the site of action, the tumor recognizable peptides, iVR1, was further decorated on the developed NPs-Apa/Sal. The iVR1 peptides was designed to specifically target the vascular endothelial growth factor receptor-1 (VEGFR-1) (Cicatiello et al., 2015). Previous study showed that iVR1 has excellent ability to inhibit the neoangiogenesis and progress of colorectal cancer by selectively antagonize the VEGFR1 (Cicatiello et al., 2015). VEGFR-1 played significant role in tumorigenesis and progress of many cancer types as well as the GC (Zhu et al., 2015; Dziobek et al., 2019), the iVR1 decorated NPs-Apa/Sal (iVR1-NPs-Apa/Sal) was therefore supposed to able of targeting deliver the loaded drugs to gastric tumor site. Our study showed the excellent tumor targeting drug delivery efficacy of iVR1-NPs-Apa/Sal *in vitro* and *vivo*, and finally resulted in a relative satisfactory anti-tumor effect. The results of this study may provide a novel strategy for combating the drug-resistance and enhancing the chemotherapy effect.

## Materials and methods

### Materials

Sal and Apa were purchased from Meilun Biotechnology (Dalian, China). Fluorescein isothiocyante (FITC) was achieved from Sigma-Aldrich (St. Louis, MO, USA). The primary and second antibodies were obtained from Abcam (Cambridge, MA, USA). The RPMI-1640 medium and fetal bovine serum (FBS) were obtained from Gibco BRL (Carlsbad, CA, USA). The 0.25% trypsin-EDTA and penicillin, and streptomycin were purchased from Invitrogen Co., USA. The Cell counting kit-8 (CCK-8) and Annexin V-FITC Apoptosis Detection kit were bought from BD PharMingen (Heidelberg, Germany). All the other solvents were of chromatographic grade and obtained from Sinopharm Chemical Reagent Co., Ltd (Shanghai, China).

### Cells and animals

The MKN-45 cell lines were obtained from the American Type Culture Collection (Manassas, VA). The cells were cultured in the RPMI-1640 medium supplemented with 10% FBS, 100 mg/ml of streptomycin, and 100 U/ml of penicillin. The drug-resistant MKN-45 cell lines (MKN-45/MDR) were established by transfection of the MKN-45 cells with HIF genes. To maintain the drug-resistance of MKN-45/MDR cells, the cells were then cultured in medium containing a low dose of Apa (0.2 µg/ml) for a week before experiments. The male BALB/c mice (20 ± 2 g) were provided by the BK Lab Anima Ltd. (Shanghai, China) and raised under the standard condition with free access to food and water. Importantly, the animal experiments preformed here were carried out in accordance with the guidelines approved by Affiliated Tumor Hospital of Zhengzhou University.

### Preparation of iVR1-NPs-Apa/Sal

The dual drugs-loaded NPs were developed by the emulsion solvent evaporation method. In brief, the blend of Sal (1 mg), Apa (1 mg), PLGA-PEG-COOH (2 mg), and PLGA-MPEG (18 mg) was mixed completely and dissolved by 1 mL dichloromethane. Then 2 mL of sodium cholate solution was supplemented followed by sonication to form the dual drugs-loaded nanoparticles (NPs-Apa/Sal). The obtained (NPs-Apa/Sal was subsequently purified by evaporation to remove the residual organic solvent and centrifugation under the condition of 14,000 rpm and 4 °C. For peptides (iVR1) modification, the collected NPs-Apa/Sal were dissolved by 2 mL PBS and reacted with iVR1 peptides under the catalytic action of NHS (100 mM) and EDC (200 mM). The FITC-labeled NPs were developed using the same method as above except that the drugs were replaced with equivalent of FITC.

### Characterization of the developed NPs-Apa/Sal and iVR1-NPs-Apa/Sal

Particle size and zeta-potential of the NPs were determined by Zetasizer Nano ZS instrument (Malvern, Worcestershire, UK). Morphology of iVR1-NPs-Apa/Sal was photographed using the transmission electron microscope (TEM) (H-600, Hitachi, Japan). Successful modification of iVR1 peptides on the surface of NPs was confirmed using the X-ray photoelectron spectroscopy (XPS) analysis (Perkin Elmer). Besides, the encapsulation efficiency (EE%) and drug loading (DL%) were further determined by high performance liquid chromatography (HPLC) and calculated with following formulas:

$$\mathrm{EE}\;\left(\%\right)=\frac{\text{Drugs in the nanoaprticles}}{\text{Total amount of drugs in dispersion}}\times 100\%$$

$$\mathrm{LC}\;\left(\%\right)=\frac{\text{Amount of drugs in nanoaprticles}}{\text{Nanoparticles weight}}\times 100\%$$

### Investigation of drug release behavior

Releases of Sal and Apa from the developed iVR1-NPs-Apa/Sal were investigated by the equilibrium dialysis method. Phosphate buffer solution (PBS, pH 7.4) containing 10% rat plasma was acted as the release media. 10 mg of NP samples were dissolved by the release medium and then loaded into a dialysis bag (MWCO = 8000 Da, Greenbird Inc., Shanghai, China). Subsequently, the NP-contained dialysis bag was immersed in 30 ml release medium followed by incubation for 72 h at 37 °C with a shaking speed of 100 rpm. 100 µL of samples were collected at different time points followed by analysis using the HPLC system.

### Cell uptake and co-localization assay

Cell uptake and co-localization assay were performed on the drug-resistant MKN-45 cell lines (MKN-45/MDR). For experiments, 5 × 103 cells were seeded into each well of the 96-well plates. After an overnight of incubation, the cells were treated with FITC-labeled various NPs with different concentrations. After different times of incubation, the cells were washed three times with PBS and fixation by 4% paraformaldehyde. Then the fluorescent signal of cells was determined by a fluorescent microscopy (Leica DMI4000 B, Germany) and quantitatively determined by the flow cytometer (FACSCalibur, BD Biosciences, USA). For co-localization assay, cells were treated as above and stained with Lysotracker Red to visualize the lysosomes. Finally, the cells were qualitatively analyzed *via* a confocal microscope (TCS SP5, Leica).

To further investigate the mechanisms of cellular uptake, the MKN-45/MDR cells were seeded as above and pretreated with various endocytic inhibitors, including the chlorpromazine (10 mg/ml), colchicines (4 mg/ml), filipin (5 mg/ml), NaN_3_ (10 mM), cyto-D (10 mg/ml), monensin (200 mM). Then the cells were, respectively, treated with FITC-labeled NPs (NPs-FITC) and iVR1-NPs (iVR1-NPs-FITC). Finally, the cells were qualitatively analyzed using the flow cytometer as above.

### Cell growth assay

Cell growth rate of the MKN-45/MDR cells were determined using the CCK-8 assay. For experiments, 5 × 103 cells were cultured in the 96-well plates and allowed to grow for overnight. Then the cells were incubated with different NP formulations for 12 h, 24 h, 36 h, and 48 h, respectively. After that, 10 µL of CCK-8 solution was added into each well of the plates and incubated for 2 h. Finally, the absorbance value of each well was examined by the microplate reader (Thermo Multiskan MK3, USA).

### Cell apoptosis assay

Cell apoptosis assay was performed on the drug-resistant MKN-45 cell lines (MKN-45/MDR). For experiments, 5 × 105 cells were seeded into each well of the 6-well plates and incubated for overnight. Then the cells were treated with various NP formulations and allowed to incubate for 12 h. After that, the treated cells in each well were collected by centrifugation (1000 g) for double-staining using the ANXA5/annexin V-FITC/PI Detection Kit (Invitrogen). Then the cell apoptosis rate was analyzed by the flow cytometry (BD FACScan Flow cytometer, USA). The cells without any treatment were used as the control.

### Migration and invasion assay

The migration and invasion ability of MKN-45/MDR cells were, respectively, evaluated by the wound healing assay and trans-well assay. For migration investigation, 5 × 103 cells were cultured in the 96-well plates and allowed to grow for 90% confluence. Then a scraped area was created using the pipette tip. The unattached cells were washed and the remaining cells were continued to culture with serum-free medium containing different formulations for 24 h. The migration rate of cells was determined by calculating the percentage of wound closure using the ImageJ software (Bethesda, MD).

To perform the trans-well experiments, 200 µL cells suspension containing 2 × 105 cells was transferred into the upper chamber of 24-well plates which was preliminary covered with Matrigel. Simultaneously, the lower chamber was filled with 200 µL of completed medium. After 24 h of incubation, the invaded cells were stained with crystal violet followed by qualitative and semi-quantitative analysis.

### Tumor targeting drug delivery efficacy of iVR1-NPs-Apa/Sal

To investigate the tumor targeting ability of iVR1-NPs-Apa/Sal *in vio*, the GC-bearing mice were developed firstly. In brief, 1 × 106 MKN-45 cells or MKN-45/MDR cells were subcutaneously injected on the right flanks of mice. Then the tumor-bearing mice were raised under the standard condition for two weeks. For tumor targeting assay, the tumor-bearing mice were randomly grouped (*n = 3*) and, respectively, treated with NPs-Apa/Sal and iVR1-NPs-Apa/Sal. 24 h later after the administration, all mice were subjected to euthanasia with main organs, including the heart, kidneys, liver, spleen, and lung were obtained as well as the tumors. Subsequently, drug concentrations of Apa and Sal in each tissues were quantitatively examined by LC-MS/MS analysis.

### Anti-tumor effect of iVR1-NPs-Apa/Sal *in vivo*

The GC-bearing mice were randomly divided into five groups (*n = 12*) and, respectively, injected with free Apa, NPs-Apa, NPs-Sal, NPs-Apa/Sal, and iVR1-NPs-Apa/Sal. The treatments were done every two weeks for three times and the dosage of Apa and Sal were both of 5 mg/kg. Then the anti-tumor effect was evaluated by detection of the tumor volume changes and medium survival time of mice in each group. Additionally, at the end of the above experiments, all tumor tissues were obtained for immunohistochemistry evaluation and western-blot analysis.

### Immunohistochemistry analysis and TUNEL assay

Tumor tissues were obtained at the end of *in vivo* tumor growth experiments and subjected to preparation of 5 µm sections. Then the tumor sections were incubated with various anti-HIF-α primary antibodies for overnight. After that, the samples were continued to incubate with streptavidin peroxidase-conjugated secondary antibodies. Finally, the results were observed by the confocal microscopy analysis (LSM710, Leica, Germany) microscope.

For TUNEL assay, the obtained tumor tissues were embedded in paraffin and then prepared for 5 μm tumor tissue sections. For cell apoptosis detection, the tumor sections were determined using the Tdt-mediated dUTP nick-end labeling (TUNEL) method.

### Western blot analysis

Protein samples were isolated by the RIPA buffer containing protease inhibitor followed by concentration detection using the bicinchoninic acid method. Then the samples were transferred to PVDF membranes after separated by 10% SDS-PAGE. After that, 5% % fat-free milk in TBST was added and incubated with samples for 1 h before interaction with various primary antibodies for overnight. For qualitative and semi-quantitative evaluation, HRP-conjugated secondary antibodies were added and incubated with samples for 1 h followed by analysis using the chemiluminescence method.

### Statistical analysis

In this study, comparisons between multiple groups were performed by the *t*-tests and ANOVAs suing the GraphPad Prism v7.0. The data were presented as means ± standard deviation (SD) with *p* < 0.05 was considered to be the threshold of significance.

## Results and discussion

### Hypoxia environment of the MKN-45 cells elevated the level of MDR1 and finally contributed to rapid tumor progress and poor survival of gastric cancer

**Figure 1.**
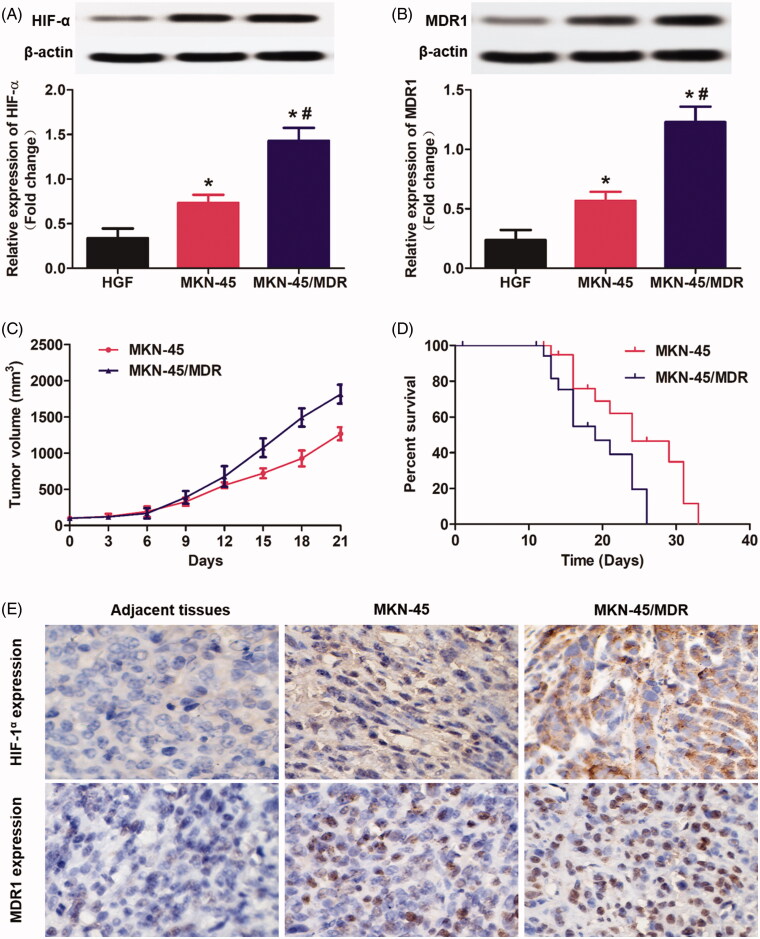
Overexpression of HIF-1α in gastric cancer cells up-regulated the MDR1 levels and finally resulted in rapid tumor progress and poor survival. (A) Determination of the HIF-1α levels in MKN-45 cells, MKN-45/MDR cells, and the control HGF cells by Western blot analysis. (B) Evaluation of the MDR1 signals in MKN-45 cells, MKN-45/MDR cells, and the control HGF cells by Western blot analysis. (C) Tumor volume changes of the mice, respectively, transplanted with MKN-45 cells and MKN-45/Apa cells. (E) Percent survival time of the MKN-45 and/or MKN-45/MDR cancer-bearing mice. *p < 0.05, significantly high than the control group. (E) Qualitative detection of HIF-1α and MDR1 signal in tumor slides by immunohistochemical assay. *p < 0.05, significantly high than the control group. ^#^*p* < 0.05, significantly high than the MKN-45 group.

Drug resistance represents one of the most intractable problem in clinical cancer chemotherapy (Chang, 2011). The ATP-driven multidrug resistance (MDR) efflux transporters, including the MDR1 and MRP, have been shown to be the primary mechanisms of chemoresistance (Aller et al., 2009). Previous studies have shown that overexpression of HIF-1α within solid tumor facilitated the expression of MDR1 and MRP by inducing the hypoxia environment (Fang et al., 2001; Comerford et al., 2002).Therefore, in this study, the drug-resistance of MKN-45/MDR cells was developed by transfection of the MKN-45 cells with HIF-1α. As shown in Figure 1(A), it was shown that the developed MKN-45/MDR cells was expressed with higher level of HIF-1α than the MKN-45 cells. Additionally, high level of HIF-1α in MKN-45/MDR cells leaded to an obvious stronger signal of MDR1 than the MKN-45 cells lack of HIF-1α (Figure 1(B)). Moreover, the levels of HIF-1α and MDR1 in both of MKN-45 cells and MKN-45/MDR cells were signally higher than the normal gastric fibroblasts HGF cells.

Subsequently, the role of HIF-1α and MDR1 in promotion of tumor progress was evaluated. As shown in Figure 1(C), the mice bearing with MKN-45 cancer displayed an obvious lower tumor growth rate than the mice bearing with MKN-45/MDR cancer. Such difference in tumor volume changes finally resulted in a significant longer medium survival time was achieved by the MKN-45 tumor-bearing mice than the MKN-45/MDR tumor-bearing mice (Figure 1(D)). At the end of tumor growth experiments, all tumor tissues were obtained for further detection of HIF-1α and MDR1. Immunohistochemical staining revealed that the tumor tissues in the group of MKN-45/MDR showed the highest level of HIF-1α and MDR1 in all groups (Figure 1(E)). Moreover, obvious stronger HIF-1α and MDR1 signal was detected in the MKN-45 group than that in the adjacent tissues. These results further confirmed the significant role of HIF-α and MDR1in promotion of tumor growth and progress.

### Characterization of nanoparticles

**Figure 2.**
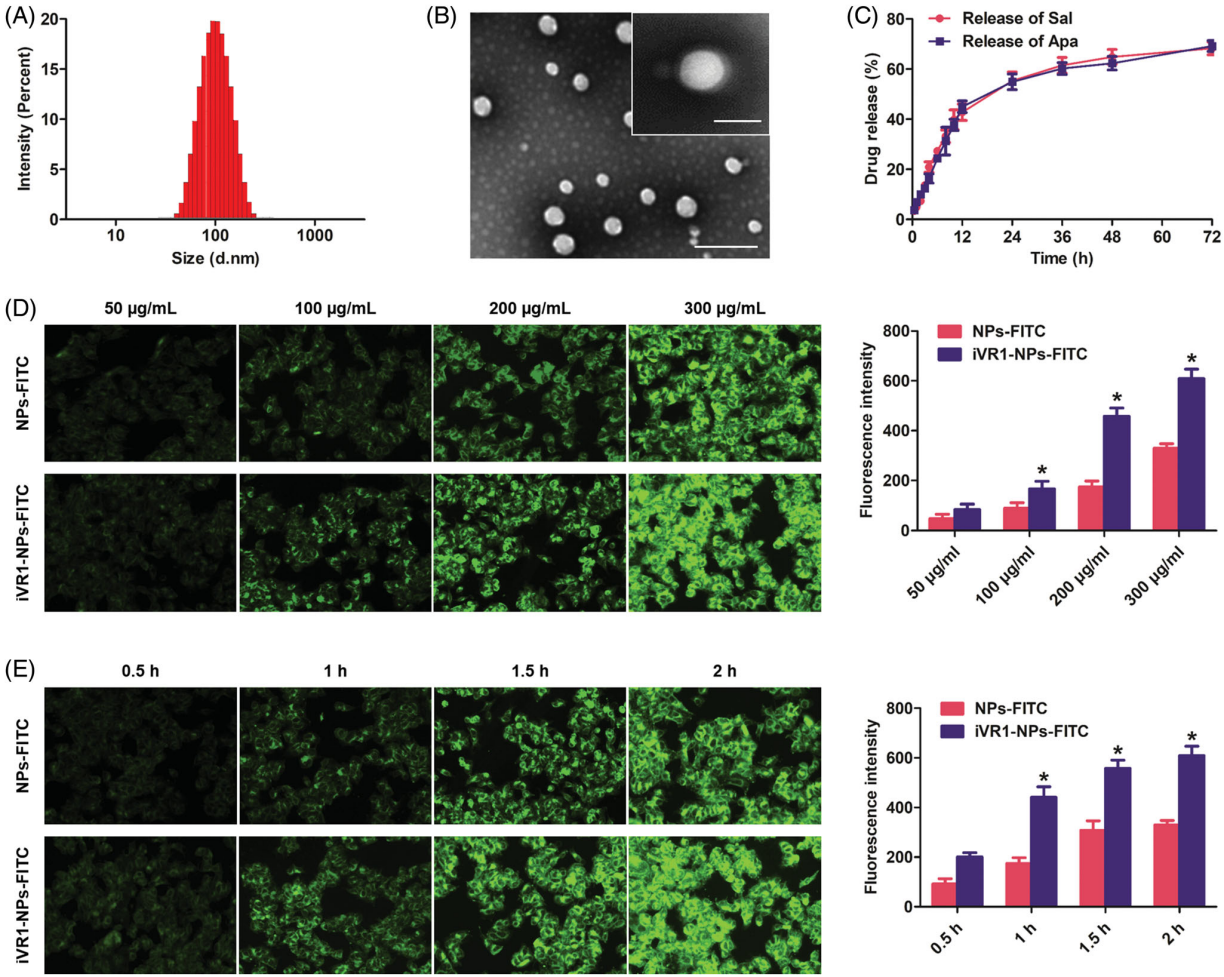
Physicochemical characterization of the developed iVR1-NPs-Apa/Sal and evaluation of tumor cells targeting ability of the iVR1-NPs-FITC *in vitro*. (A) Particle size and size distribution of iVR1-NPs-Apa/Sal. (B) Morphology of iVR1-NPs-Apa/Sal determined by TEM. (C) Release of Sal and Apa from iVR1-NPs-Apa/Sal in the medium of PBS supplemented with 10% rat plasma. (D) Qualitative and quantitative evaluation of cell uptake after incubation of cells with difference concentration of nanoparticles. (E) Qualitative and quantitative evaluation of cell uptake after incubation of cells with nanoparticles for different times. *p < 0.05, significantly higher than the NPs-FITC treated cells.

Besides the tumor biology and patient characteristics, the delivery efficacy of drugs to tumor site by NPs is also signally affected by a wide range of physicochemical properties, especially the particle size, morphalogy, and surface charge (Ernsting et al., 2013). In terms of the particle size, it was widely accepted that 100 nm around was the optimal diameter that leveraging the EPR effect and maximizing the drug delivery efficacy (Kulkarni and Feng, 2013). In our study, the developed iVR1-NPs-Apa/Sal showed an average size of 104.32 nm and showed a relative narrow size distribution (Figure 2(A)). Besides, morphology of the iVR1-NPs-Apa/Sal obtained by TEM showed a nearly spheroid shape and an obvious core-shell structure (Figure 2(B)). Modification of iVR1 was confirmed by the XPS assay and results revealed that the surface nitrogen detected on iVR1-NPs-Apa/Sal was 0.63%. However, the signal of nitrogen detected on the surface of NPs-Apa/Sal was negligible. Drug loading capacity and encapsulation efficacy were further determined. The EE (%) for Sal and Apa was 46.31% and 48.52%, respectively, while the LC (%) for Sal and Apa was 1.03% and 0.94%, respectively. Drug release investigation displayed a similar and controlled release pattern for Sal and Apa, with just 65% around of drugs was released at 72 h (Figure 2(C)).

### Cell uptake of nanoparticles

Cell uptake experiments were performed by incubation of cells with different concentration of NPs and for different times. As shown in Figure 2(D), cells treated with iVR1-NPs-FITC displayed the obvious higher level of internalization compared with the cells incubated with NPs-FITC. Moreover, MKN-45/MDR cells uptake of NPs was concentration-dependent and the biggest difference between NPs-FITC and iVR1-NPs-FITC was obtained when the concentration of NPs was set at 200 µg/mL. Consistent with the above results, it was also observed that MKN-45/MDR cells uptake of NPs was time-dependent (Figure 2(E)). Further quantitative analysis

**Figure 3.**
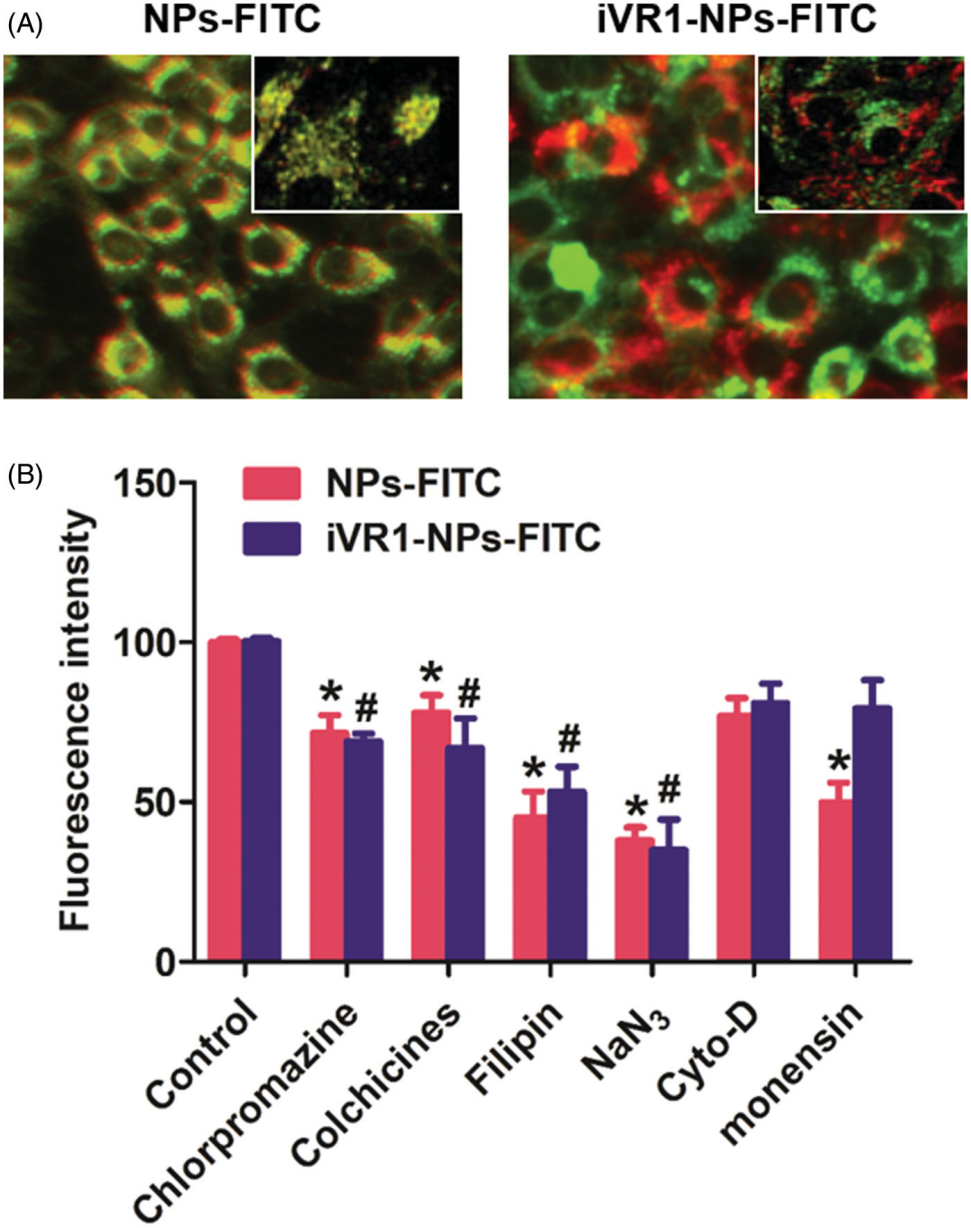
Evaluation of the intracellular nanoparticles distribution and cell uptake mechanisms. (A) Qualitative evaluation of the co-localization of cells with nanoparticles. The green represent nanoparticles and red represents the lysosomes. (B) Cellular uptake of NPs-FITC and iVR1-NPs-FITC in the presence of various endocytosis inhibitors: chlorpromazine (10 mg/ml), colchicines (4 mg/ml), filipin (5 mg/ml), NaN3 (10 mM), cyto-D (10 mg/ml), monensin (200 mM). *p < 0.05 and #*p* < 0.05 signally different from the control group.

Intracellular NP distribution was further investigate by the co-localization assay. As shown in Figure 3(A), it was displayed that the lysosomes was involved in the cell uptake of iVR1-NPs-FITC and NPs-FITC. Of great importance, most of NPs-FITC while not the iVR1-NPs-FITC was co-localized with lysosomes after 1 h of incubation, suggested that the iVR1-NPs-FITC was able of mediating more NPs escape from the capture of lysosomes. Such results could be further confirmed by the cellular mechanisms study. As shown in Figure 3(B), pretreated the MKN-45/MDR cells with monensin (lysosome inhibitor) could signally restricted cellular uptake of the NPs-FITC while not the iVR1-NPs-FITC, suggested that cell uptake of NPs-FITC was lysosomes-mediated. Moreover, cell uptake of NPs-FITC and iVR1-NPs-FITC were both dramatically inhibited by the chlorpromazine (clathrin-mediated endocytosis pathway inhibitor), colchicine (microtubule depolymerization agent), filipin (caveolae-mediated endocytosis inhibitor), and NaN_3_ (energy-depletion agent) while not the Cyto-D (actin polymerization disrupting agent).

### The iVR1-NPs-Apa/Sal showed excellent anti-tumor effect *in vitro*

**Figure 4.**
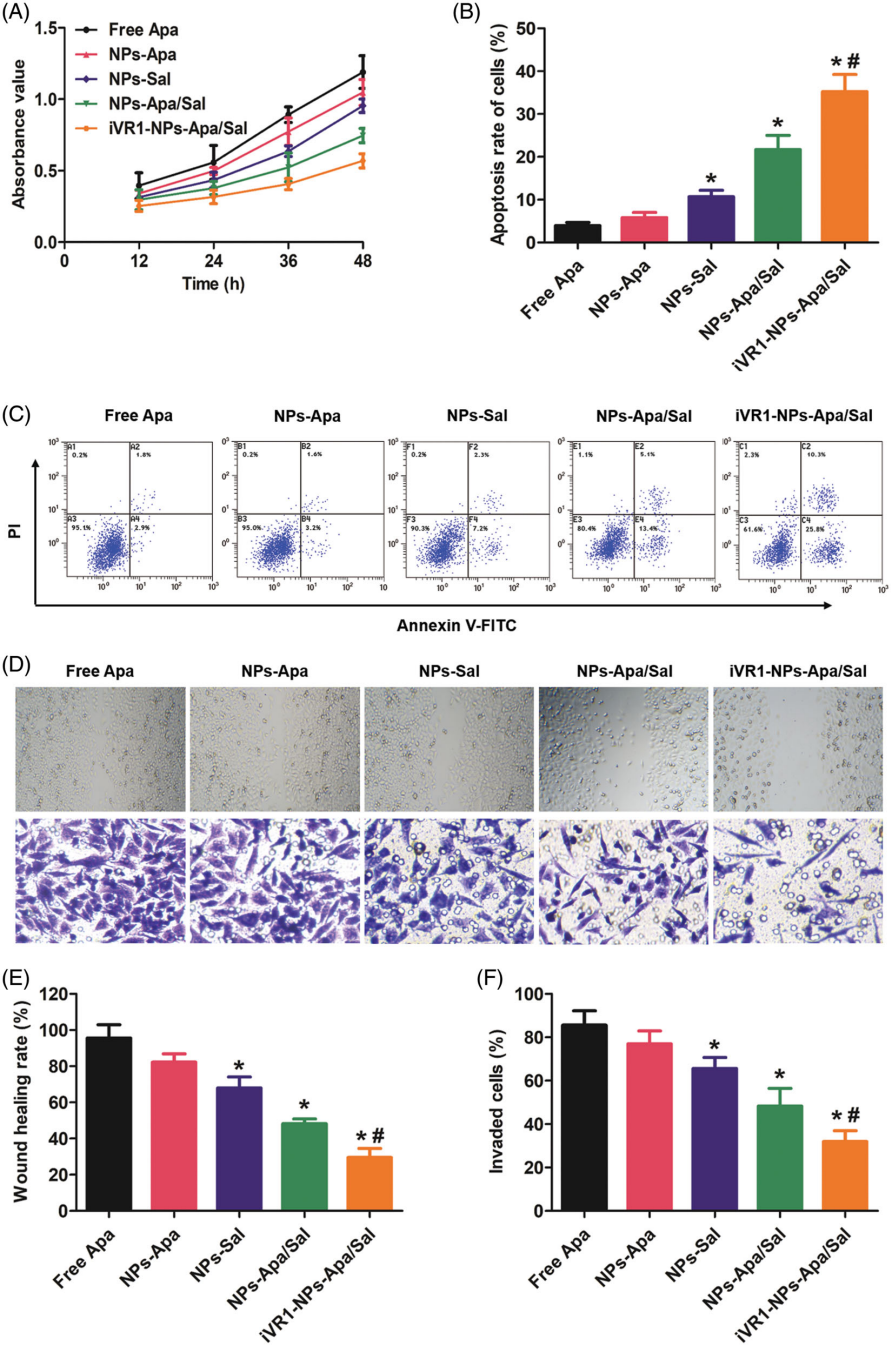
Evaluation of anti-tumor effect of iVR1-NP-Apa/Sal *in vitro*. (A) Cell growth rate of the MKN45/MDR cells after various treatments determined by evaluation of the absorbance values using the CCK-8 method. (B) Quantitative analysis of the cell apoptosis rate after treated with different nanoparticle formulations by the Annexin v-FITC/PI double staining method. (C) Qualitative evaluation of the cell apoptosis rate after various treatments through the Annexin v-FITC/PI double staining approach. (D) Cell migration rate and invasion rate were, respectively, investigated by the wound healing assay (the figures above) and trans-well experiment (the figures below). Quantitative analysis the migration rate (E) and invasion rate (F) of MKN-45/MDR cells after treated by different strategies. *p < 0.05 signally different from the group of Free Apa. #*p* < 0.05 signally different from the group of NPs-Apa/Sal.

Cell growth rate was determined by examination of the absorbance value after various treatments. As shown in Figure 4(A), cells in the group of NPs-Apa showed significant higher value than the cells in NP-Sal group. However, the cells incubated with NPs-Apa/Sal displayed obvious lower absorbance value than the cells incubated with NPs-Apa or NPs-Sal, suggested distinct advantage of the combination therapy strategy. Moreover, decoration of the NPs-Apa/Sal with iVR1 peptides resulted further down-regulation of the absorbance value. Cell apoptosis assay was subsequently performed followed by qualitative and quantitative analysis. As shown in Figure 4(B) and 4(C), the results indicated that the cells treated by iVR1-NPs-Apa/Sal showed the highest rate of cell apoptosis among all groups. Moreover, similar to the cell growth assay, cells treated by NPs-Apa/Sal displayed obvious stronger cell apoptosis rate than the cells only treated by NPs-Sal or NPs-Apa.

To investigate the metastatic potential of MKN-45/MDR caner *in vitro*, the wound healing assay and trans-well assay was, respectively, applied to evaluate the migration and invasion ability of the MKN-45/MDR cells. As shown in Figures 4(D), 3(F), there was no significant difference was observed between the group of control and PLGANP-Apa while obvious lower migration and invasion rate was detected in the PLGANP-Sal group. Moreover, cells treated by NP-Apa/Sal resulted stronger ability of inhibition on cell migration and invasion than the cells only received with monotherapy and the antagonism effect was dramatically enhanced after decoration with iVR1 peptides.

### Sal enhanced the chemotherapy effect of apa through inducing cell apoptosis and reprograming the tumor hypoxia environment

**Figure 5.**
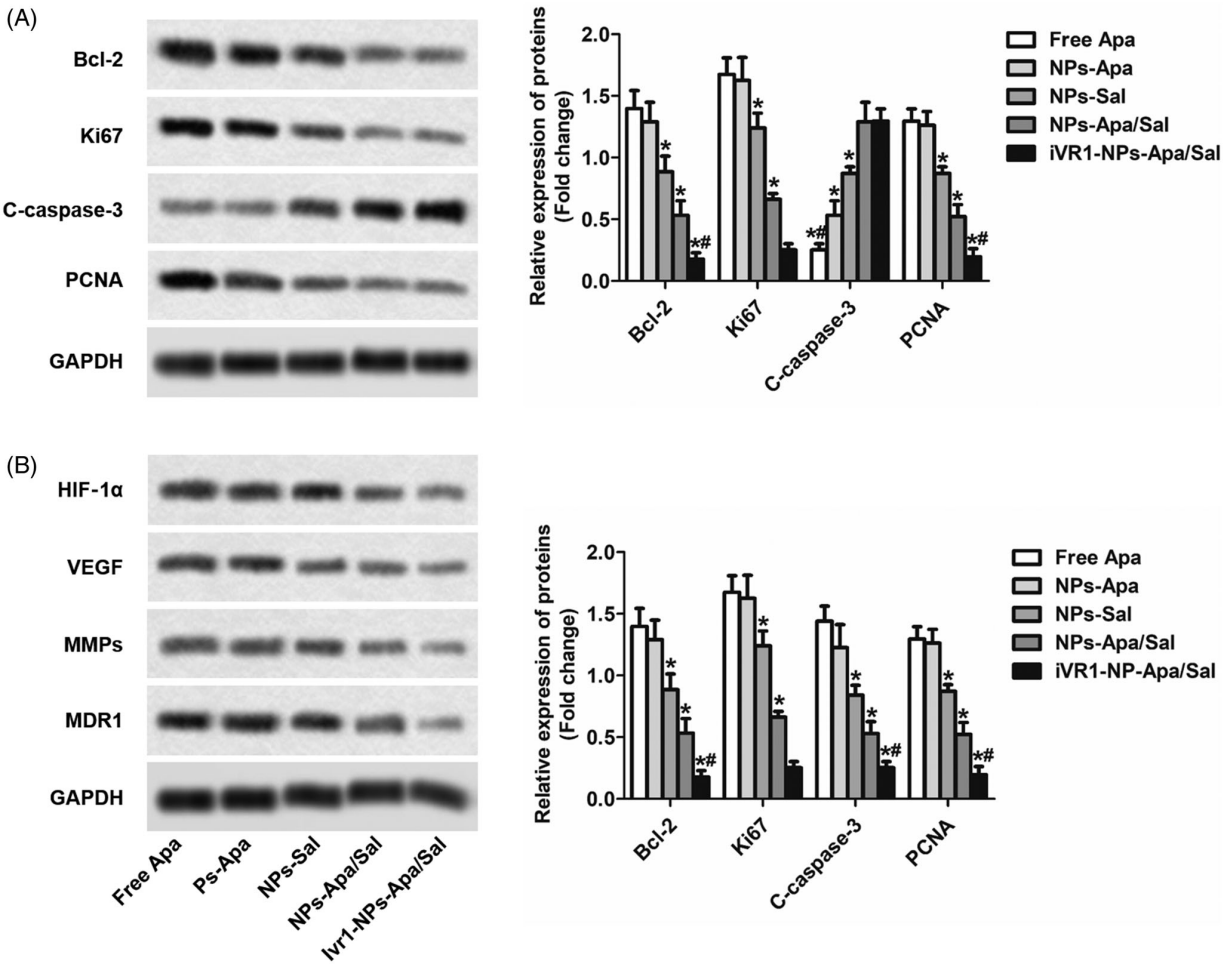
Sal enhanced the chemotherapy effect of Apa through inducing cell apoptosis and reprograming the tumor hypoxia environment. (A) The levels of pro-apoptotic factors Bcl-2 and C-caspase-3, and proliferation factors ki67 and PCNA in MKN-45/MDR cells after various treatments determined by Western-blot analysis. (B) The expression of HIF-1α, VEGF, MMPs, and MDR1 post different treatments were investigated by Western-blot assay. *p < 0.05, significantly lower than the cells treated with free Apa. #*p* < 0.05, significantly lower than the cells treated with NPs-Sa.

After the above cellular experiments, the cells after various treatments were collected for further analysis at molecular level. As shown in Figure 5(A), the MKN-45/MDR cells treated with NPs-Apa/Sal showed obvious lower expression of Bcl-2, ki67 and PCNA than the cells incubated with NPs-Sal or NPs-Apa. In contrast, treated the cells with NPs-Apa/Sal while not the NPs-Sal or NPs-Apa resulted in significant up-regulation of pro-apoptotic factors levels. Moreover, after decoration with iVR1 peptides, the effect of NPs-Apa/Sal induce cell apoptosis was dramatically further enhanced. The role of iVR1-NPs-Apa/Sal in regulation of the hypoxic microenvironment was further investigated. As shown in Figure 5(B), the cells incubated with iVR1-NPs-Apa/Sal displayed the lowest level of HIF-1α among all groups. Besides, treated the cells with iVR1-NPs-Apa/Sal leaded to markedly decreased expression of the VEGF, MMPs, and MDR1. These results together suggesting that the developed iVR1-NPs-Apa/Sal was able of enhancing chemotherapy effect by regulation of the tumor hypoxia micro-environment and induce cell apoptosis.

### Tumor targeting *in vivo*

**Figure 6.**
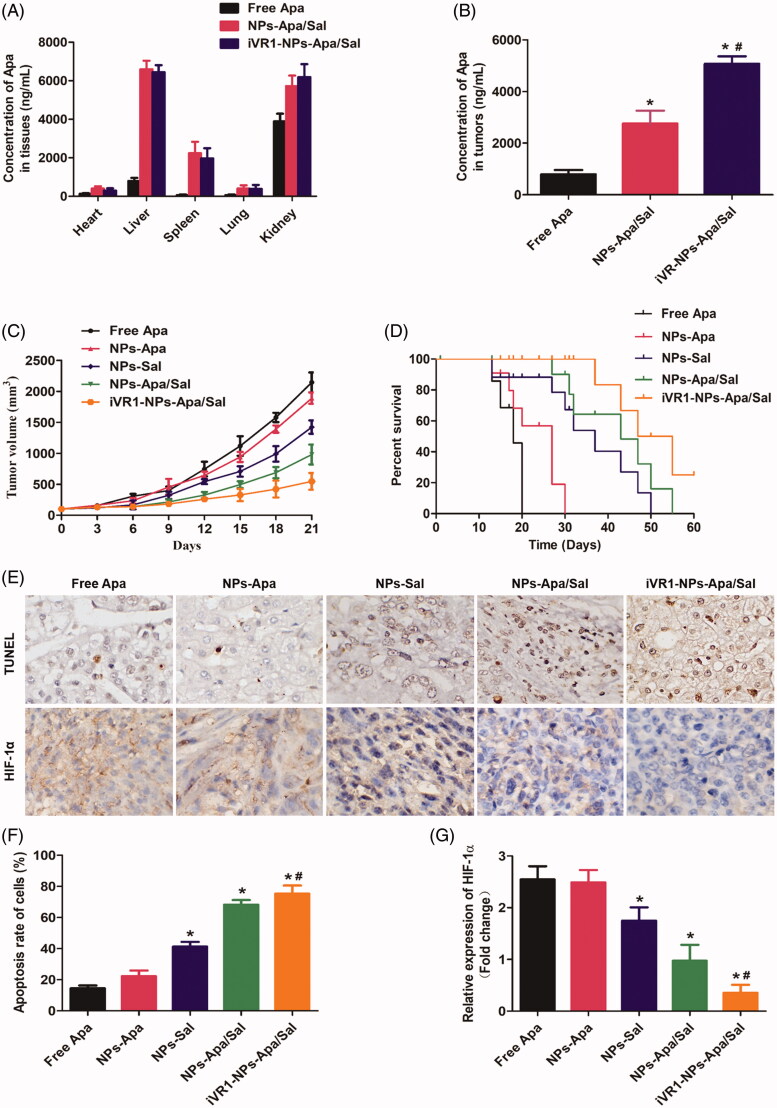
Evaluation of the tumor targeting drug delivery efficacy and anti-tumor effect of iVR1-NPs-Apa/Sal *in vivo*. (A) Bio-distribution of Apa in primary normal organs, including the heart, liver, spleen, lung, and kidney). (B) Distribution of Apa in tumor tissues of the MKN-45/MDR cancer bearing mice after, respectively, injected by free Apa, NPs-Apa/Sal, and iVR1-NPs-Apa/Sal. (C) Tumor volume changes of the tumor-bearing mice after, respectively, treated with free Apa, NPs-Apa, NPs-Sal, NPs-Apa/Sal, and iVR1-NPs-Apa/Sal. (D) Kaplan − Meier survival curve of the tumor-bearing mice after received with different treatments. (E) TUNEL detection of tumor slides and immunohistochemical analysis of the HIF-1α expression. (F) Quantitative analysis of the cell apoptosis rate in tumor sections after different treatments. (G) Quantitative evaluation of the HIF-1α levels in tumor slide after various treatments. *p < 0.05 signally different from the group of Free Apa. #*p* < 0.05 signally different from the group of NPs-Apa/Sal.

Delivery of drugs to the tumor site by NPs is severely impacted by a wide range of obstacles, such as insufficient tumor affinity, dense stroma in tumor tissues, and the renal filtration and the reticuloendothelial system (Ernsting et al., 2013; Kulkarni and Feng, 2013; Maurizi et al., 2015). It has been shown that the iVR1 peptides has high affinity to multiple tumor types including the GC (Cicatiello et al., 2015). Based on this, the iVR1 peptides were decorated on the surface of NPs-Apa/Sal for tumor targeting drug delivery. To confirm the tumor-targeting drug delivery efficacy of the developed iVR1-NPs-Apa/Sal, bio-distribution of drugs were determined. As shown in Figure 6(A), the drug concentrations in normal tissues from the NPs-Apa/Sal group were signally higher than that of the iVR1-NP-Apa/Sal group. In contrast, the mice injected with NPs-Apa/Sal resulted obvious lower drug concentrations in tumor tissues than the iVR1-NPs-Apa/Sal (Figure 6(B)). These results indicated the iVR1 peptides decorated NPs have excellent ability of tumor targeting drug delivery.

### Anti-tumor effect *in vivo*

Anti-tumor effect of iVR1-NPs-Apa/Sal *in vivo* was evaluated by determination of the tumor growth rate and percent survival time of the MKN-45/MDR tumor bearing mice. As shown in Figure 6(C,D), the mice treated by iVR1-NPs-Apa/Sal obtained the lowest tumor growth rate and the longest percent survival time among all groups. Additionally, the mice injected with NPs-Sal displayed obvious stronger antagonism effect than the control group. In contrast, negligible anti-tumor effect was achieved by NPs-Apa. However, the mice in the group of NPs-Apa/Sal showed distinct stronger anti-tumor effect than the mice treated by NPs-Sal or NPs-Apa, indicated that combination therapy of Sal and Apa has prominent advantage than the monotherapy of Sal and Apa.

After the above experiments, all of the mice were euthanasized with tumor tissues were obtained for immunohistochemical analysis of the cell apoptosis rate and HIF-1 expression. As shown in Figure 6(E–G), the MKN-45/MDR tumor-bearing mice treated with iVR1-NPs-Apa/Sal leaded to the biggest area of cell apoptosis rate than other groups. Besides, the mice treated by NPs-Apa/Sal showed obvious larger area of cell apoptosis in tumor tissues than the mice treated by NPs-Sal or NPs-Apa, suggesting an excellent anti-tumor effect of the combination therapy strategy. For evaluation of the hypoxia-inducible factor expression, it was confirmed that high levels of HIF-1α within the MKN-45/MDR tumor tissues could be signally alleviated by treating with NPs-Apa/Sal while not the NPs-Sal only. Moreover, after decoration on the surface of NPs-Apa/Sal with iVR1 peptides, the alleviation effect could be signally enhanced. Taking these results together, it could be concluded that the developed iVR1-NPs-Apa/Sal holds great potential in the improvement of anti-tumor effect on drug resistant GC by reprograming the tumor hypoxia micro-environment and induce cell apoptosis.

## Conclusion

In summary, we have developed a novel combination therapy strategy for combating the drug-resistance of GC *in vitro* and *in vivo*. To achieve that goals, the NPs-Apa/Sal was developed firstly followed by functionalization with tumor-homing peptides iVR1 (iVR1-NPs-Apa/Sal). In this study, it was shown that up-regulation of hypoxia-inducible factor resulted in elevation of MDR1 and rapid tumor progress. Subsequently, it was further confirmed that the developed iVR1-NPs-Apa/Sal have excellent capacity of tumor targeting drug delivery and resulted in more efficient inhibition effect on cell growth, migration, and invasion, and as well as the tumor progress *in vivo*. Of great importance, the obtained results revealed that treatment of the drug-resistant GC with Apa plus Sal showed obvious stronger anti-tumor effect than monotherapy of Apa or Sal. Mechanisms study showed that the Sal was able of enhancing the chemotherapy effect of Apa through inducing cell apoptosis by increase of pro-apoptotic factors and reprograming the hypoxia tumor-environment by down-regulation of hypoxia-inducible factor (HIF-1α). In a word, the developed tumor recognizable iVR1-NPs-Apa/Sal showed excellent anti-tumor effect *in vitro* and *in vivo* through inducing cell apoptosis and reprograming the tumor microenvironment and holds great potential in treating a wide range of malignant cancer types.
